# Complete mitochondrial genome of large dragonfly (*Macromia amphigena*)

**DOI:** 10.1080/23802359.2022.2039082

**Published:** 2022-02-15

**Authors:** Chae-Hui An, Kyeong-Sik Cheon, Ji-Eun Jang, Jun-Kil Choi, Hwang-Goo Lee

**Affiliations:** Department of Biological Science, Sangji University, Wonju, South Korea

**Keywords:** *Macromia amphigena*, complete mitochondrial genome, phylogeny

## Abstract

The complete mitochondrial genome of *Macromia amphigena* (Odonata; Macromiidae; Macromia) was sequenced and found to be 15,594 bp in length including 37 genes (thirteen protein-coding genes (PCGs), 22 transfer RNAs (tRNAs) and two ribosomal RNAs (rRNAs) and a non-coding region). The overall GC content of the mitochondrial genome for *M. amphigena* was 28.4%. A phylogenetic analysis conducted for 13 species within the order Odonata suggested that *Macromia daimoji* is the most closely related to *M. amphigena*.

Dragonflies belonging to the family Macromiidae are composed of 125 species and are widely distributed worldwide (Dijkstra et al. 2013). *Macromia amphigena* Selys 1,871 (Odonata, Macromiidae) had been classified as a genus of *Macromia* (Lieftinck 1955). *Macromia amphigena* is widespread primarily inhabiting middle and upstream areas in Korea, Japan and China. We report the complete mitochondrial genome sequence of *M. amphigena* to provide genetic information that can be used for various studies in the future. In addition, the relationship with *Macromia daimoji*, which was registered as an endangered species in South Korea (Jeong 2012), is unclear. For this reason, we attempt here to report the relationship.

In this study, we report for the first time the complete mitochondrial genome (mitogenome) sequence of *M. amphigena* and determine its phylogenetic position in reference to 13 other species. We did not collect material from any privately owned or protected area that required permission. Ethics approval for this study was obtained from the institutional Ethics Committee of Sangji Universtiy. The specimen of *M. amphigena* used for this study (voucher number: SJAEMA001; HG Lee, morningdew@sangji.ac.kr) was collected from Jukgyecheon (Gohyeon-dong, Yeongju-si, Gyeongsangbuk-do province; 36°50′50.33″N, 128°36′25.19″E) and stored at the Department of Biological Science of Sangji University in Korea. Total genomic DNA was extracted using a DNeasy Blood & Tissue Kit (Qiagen, Hilden, Germany). Paired-end sequencing for the mitogenome of *M. amphigena* was performed on the MiSeq (Illumina Inc., San Diego, CA) platform. We obtained 2,157,479 raw read pairs with a read length of 301 bp. The assembly and annotation of the mitogenome were accomplished using Geneious prime 2021.1.1 (Biomatters Ltd, Auckland, New Zealand). We also  compared each gene to the previously published mitochondrial genome of *M. daimoji* (NC_041425), which was suggested to be the most closely related species (Kim et al. 2018), for correct gene annotation.

The assembled mitogenome of *M. amphigena* is available at the NCBI GenBank database under the accession numbers MZ504971. The assembled mitogenome of *M. amphigena* showed a length of 15,594 bp and an overall GC content of 28.4%, long with a total nucleotide composition of A − 39.0%, C − 16.7%, G − 11.7%, and T − 32.6%. The mitogenome consists of 13 protein-coding genes (PCGs), 22 tRNA genes, and two ribosomal RNA (rRNA) genes. The structure of the PCGs in *M. amphigena* was marginarly identical to that in *M. daimoji* (NC_041425), in detail, the nt identity between them was 84.4%.

Phylogenetic analyses were done using 13 PCGs and two rRNA genes (13,924bp) from 11 species (including the species in this study) and two outgroups (*Isonychia ignota* and *Ephemera orientalis*). The sequences were aligned using MAFFT (Katoh et al. 2002) and phylogenetic trees were then constructed by maximum likelihood (ML) and maximum parsimony (MP) methods using MEGA X (Kumar et al. 2018) with 1,000 bootstrap replications. The two independent phylogenetic trees yielded the same topology. *Macromia amphigena* formed a monophyly with *M. daimoji* with a high support value (BS = 100) (Figure 1).

**Figure 1.**
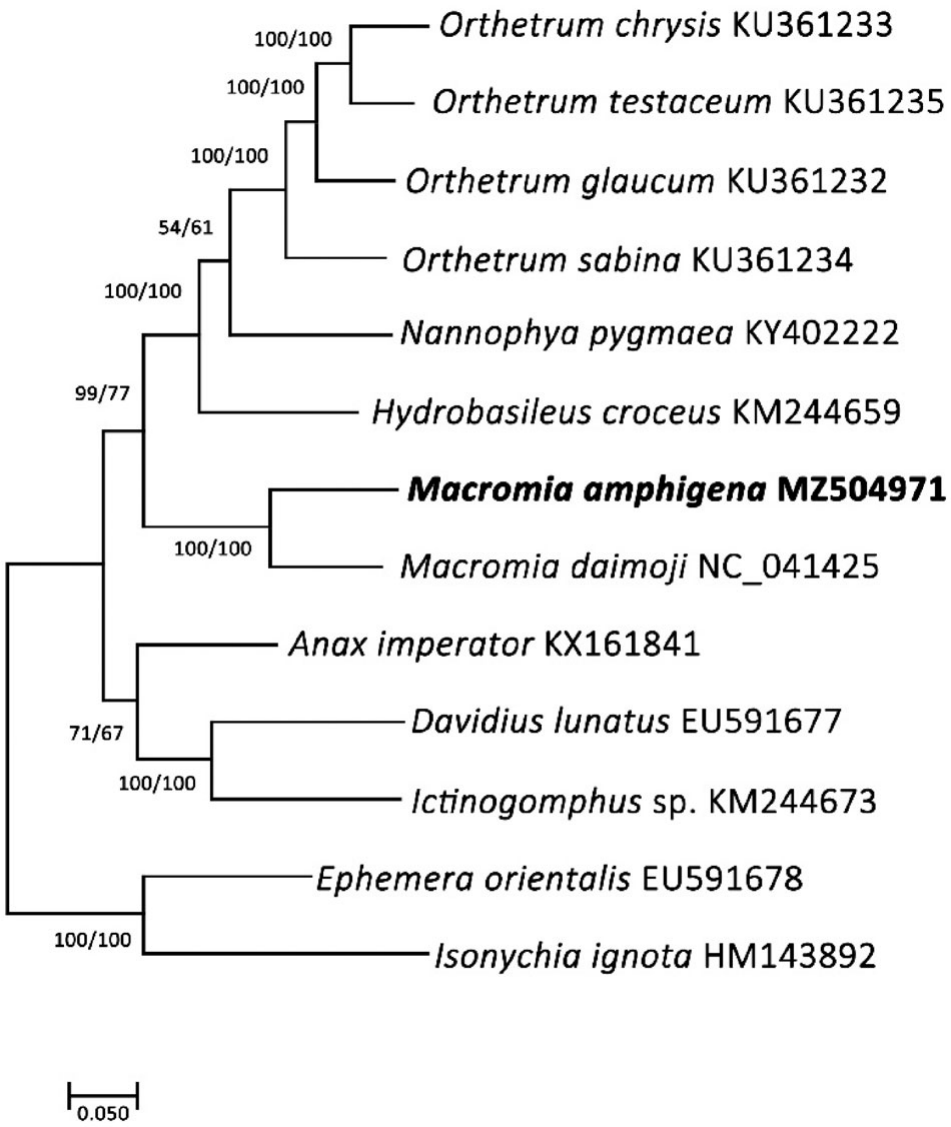
Phylogenetic tree of 13 order Odonata including *M. amphigena*. Reconstruction of maximum likelihood (ML) and maximum parsimony (MP) trees was based on 13 PCGs and two rRNA genes (13,924 bp). Numbers at the branches represent the bootstrap support values for ML (left) and MP (right), respectively. Branching patterns and branch lengths follow the results of ML analysis.

## Data Availability

The genome sequence data that support the findings of this study are openly available in GenBank of NCBI at https://www.ncbi.nlm.nih.gov. under the accession no. MZ504971. The associated BioProject, SRA, and Bio-Sample numbers are PRJNA743726, SRR15041083 and SAMN20058986, respectively.
